# Urinary vitamin D binding protein levels in children with idiopathic nephrotic syndrome: a biomarker differentiating steroid sensitive from steroid resistant nephrotic syndrome

**DOI:** 10.1590/2175-8239-JBN-2025-0094en

**Published:** 2025-11-07

**Authors:** Anup K. Chaudhary, Om P. Mishra, Ketki Khandhadiya, Surendra P. Mishra, Akash Mishra, Ashok Singh, Priyanka Dua

**Affiliations:** 1 Heritage Institute of Medical Sciences, Department of Pediatrics, Varanasi, India.; 2 Heritage Institute of Medical Sciences, Department of Biochemistry, Varanasi, India.; 3 Banaras Hindu University, Institute of Medical Sciences, Department of Biochemistry, Varanasi, India.; 4 Banaras Hindu University, Institute of Medical Sciences, Centre of Biostatistics, Varanasi, India.

**Keywords:** Childhood Idiopathic Nephrotic Syndrome, Corticosteroid Sensitive Nephrotic Syndrome, Steroid-Resistant Nephrotic Syndrome, Urinary Vitamin D Binding Protein

## Abstract

**Introduction:**

The primary objective of this study was to assess urinary vitamin D binding protein (uVDBP) levels in idiopathic nephrotic syndrome. Secondary objectives were to evaluate the ability of uVDBP to differentiate between steroid resistant nephrotic syndrome (SRNS) and steroid sensitive nephrotic syndrome (SSNS), and between minimal change disease (MCD) and focal segmental glomerulosclerosis (FSGS) of SRNS.

**Methods:**

Sixty-three cases of idiopathic nephrotic syndrome were included; 23 were first episode nephrotic syndrome (FENS), 24 were relapsing nephrotic syndrome (RNS), and 16 were steroid resistant. Eleven subjects were used as controls and 17 cases were under remission. ELISA kit was used to assay uVDBP.

**Results:**

Median uVDBP levels were increased across FENS, RNS, SRNS groups as compared to controls (P < 0.001). Median uVDBP was significantly higher in SRNS (32.2 ng/mL) than in SSNS (4.0 ng/mL, P < 0.001). Similarly, the median level was also significantly higher in FSGS (56.6 ng/mL) than in MCD (20.8 ng/mL, P = 0.005). The uVDBP was significantly lower during remission (1.2 ng/mL) than in the active (5.1 ng/mL) phase of the disease (P < 0.001). At the 12-ng/mL cut-off value, uVDBP had higher sensitivity, specificity, and area under the curve (100%, 97.9% and 0.983, respectively) for distinguishing steroid-resistant from steroid-sensitive patients. Significant positive correlation was found between uVDBP and urine protein/creatinine ratio (r = 0.441, P < 0.001) and negative correlation between uVDBP and serum albumin (r = −0.303, P = 0.019).

**Conclusion:**

uVDBP level is related to disease activity and can distinguish not only steroid-resistant from steroid-sensitive, but also FSGS from MCD histological subtypes of steroid-resistant NS.

## Introduction

Nephrotic syndrome is a common glomerular disorder, with the annual incidence being 2 to 7 /100,000 in children below 16 years of age^1^. The Asian population has a higher incidence (9–16 per 100,000 children per year)^2^. The majority (85–90%) of cases of idiopathic nephrotic syndrome has a favorable response to corticosteroid treatment, being categorized as steroid-sensitive nephrotic syndrome (SSNS). However, 10–15% of the cases do not achieve remission with steroid treatment and are classified as steroid-resistant nephrotic syndrome (SRNS)^3^, with a significant proportion (30–50%) progressing to end-stage kidney disease within 10 years^4,5^. Urinary biomarkers like urinary N-acetyl-beta D glucosaminidase activity^6^, neutrophil gelatinase-associated lipocalin^7^–^9^, and CD 80^10,11^ have been used in children with idiopathic nephrotic syndrome to distinguish between steroid-sensitive and resistant forms of the disease. However, sensitivity and specificity for the differentiating ability of such biomarkers vary among studies. The levels also vary between two common histopathological subtypes of SRNS: minimal change disease (MCD) and focal segmental glomerulosclerosis (FSGS)^8,12^.

The vitamin D binding protein (VDBP) is primarily produced in the liver. The molecular weight of VDBP is similar to that of albumin and, therefore, it is excreted in urine along with albumin in patients with nephrotic syndrome. Cases of SRNS have heavy proteinuria and protracted course and can have higher excretion of VDBP in urine than SSNS. Kidney biopsy is an invasive procedure required to diagnose FSGS and MCD, which have different steroid response and long-term outcomes.

The levels of uVDBP can also vary between the two subtypes, with FSGS cases having more severe proteinuria than MCD^8^. Therefore, VDBP, which can be measured by a non-invasive test, might serve as a biomarker to differentiate steroid-resistant from steroid-sensitive patients, and between biopsy-proven FSGS and MCD of SRNS. This study aimed to estimate uVDBP levels in idiopathic nephrotic syndrome and analyze its ability to distinguish between SRNS and SSNS, and between FSGS and MCD cases.

## Methods

This was a prospective study carried out between December 2022 and June 2024 at a teaching hospital. The study was approved by the Institutional Ethics Committee (HIMS/IEC/111/2022, dated 18.11.2022). Parents/legal guardians provided written informed consent before enrolment in the study.

### Study Subjects

Patients of both genders with idiopathic nephrotic syndrome (INS) aged 1–18 years were enrolled. The diagnostic criteria were presence of edema, proteinuria [urine protein/creatinine ratio (uPr/Cr) of >2 mg/mg)] and decreased serum albumin level (serum albumin <3g/dL). The exclusion criteria were presence of congenital or infantile nephrotic syndrome, concomitant urinary tract infection, nephrotic syndrome of secondary etiologies, acute kidney injury or chronic kidney disease, congenital malformations of kidney and urinary tract, and kidney transplantation.

Sample size calculation was based on the study of Choudhary et al.^13^ on uVDBP in patients with nephrotic syndrome. The mean and SD values were 701.12 ± 372.0, 252.87 ± 66.34, and 34.7 ± 14.1 ng/mL in SRNS, SSNS, and controls, respectively. Considering a minimum difference in the mean to be detected between the groups of 218.1 ng/mL, a maximum SD of 372, a 5% significance level, and 90% power, the minimum number of cases to be included in each group was found to be 27. Thus, total number of study subjects required was 81. Considering a 10% dropout rate, the total sample size calculated was 81 + 8.1 = 89.1, which was rounded to 90.

Sixty-three cases among different categories of INS were included: SSNS [23 first episode NS (FENS) and 24 relapsing nephrotic syndrome (RNS: 5 infrequent relapsers, 9 frequent relapsers, and 10 steroid dependent] and 16 SRNS. Another 17 children [13 SSNS, (2 FENS + 11 RNS) and 4 SRNS] were also included during remission (urine dipstick- nil/trace and/or uPr/Cr ratio <0.2 mg/mg for 3 consecutive days). Eleven normal children in the same age group were selected to serve as controls (the number of total study subjects was 63 + 17 + 11 = 91).

### Urinary Vitamin D Binding Protein (Uvdbp) Assay

Urine samples were collected from patients during the active (proteinuric) phase of the disease in a sterile container, centrifuged at 5000 rpm for 5 min, and stored at –20°C until assayed. The concentration of uVDBP was estimated by using commercially available Human VDBP ELISA kit, manufactured by ELK (Wuhan) Biotechnology Co. Ltd. (http://www.elkbiotech.com). The kit sensitivity was 0.074 ng/mL (reference range detection of 0.16–10 ng/mL, intra-assay precision <8% and inter-assay precision <10%).

### Statistical Analysis

SPSS version 29.0.2.0. (20) was used for data analysis. Fisher exact test was applied to compare categorical variables. Analysis of variance (ANOVA) was used to compare variables with normal distribution, and Kruskal Wallis test was applied to compare data that did not follow a Gaussian distribution. Bonferroni test was used for post-hoc analysis and Mann Whitney U test was used for two-group comparisons. Paired Student’s t and Wilcoxon signed rank tests were used for paired sample observations, depending on the data distribution. To find out the sensitivity, specificity, and area under the curve of uVDBP in distinguishing SRNS from SSNS, the receiver operating characteristic (ROC) curve was generated. Spearman’s rho correlation coefficients were obtained for uVDBP in relation to different variables, and p value of <0.05 was considered significant.

## Results

**Figure 1:**
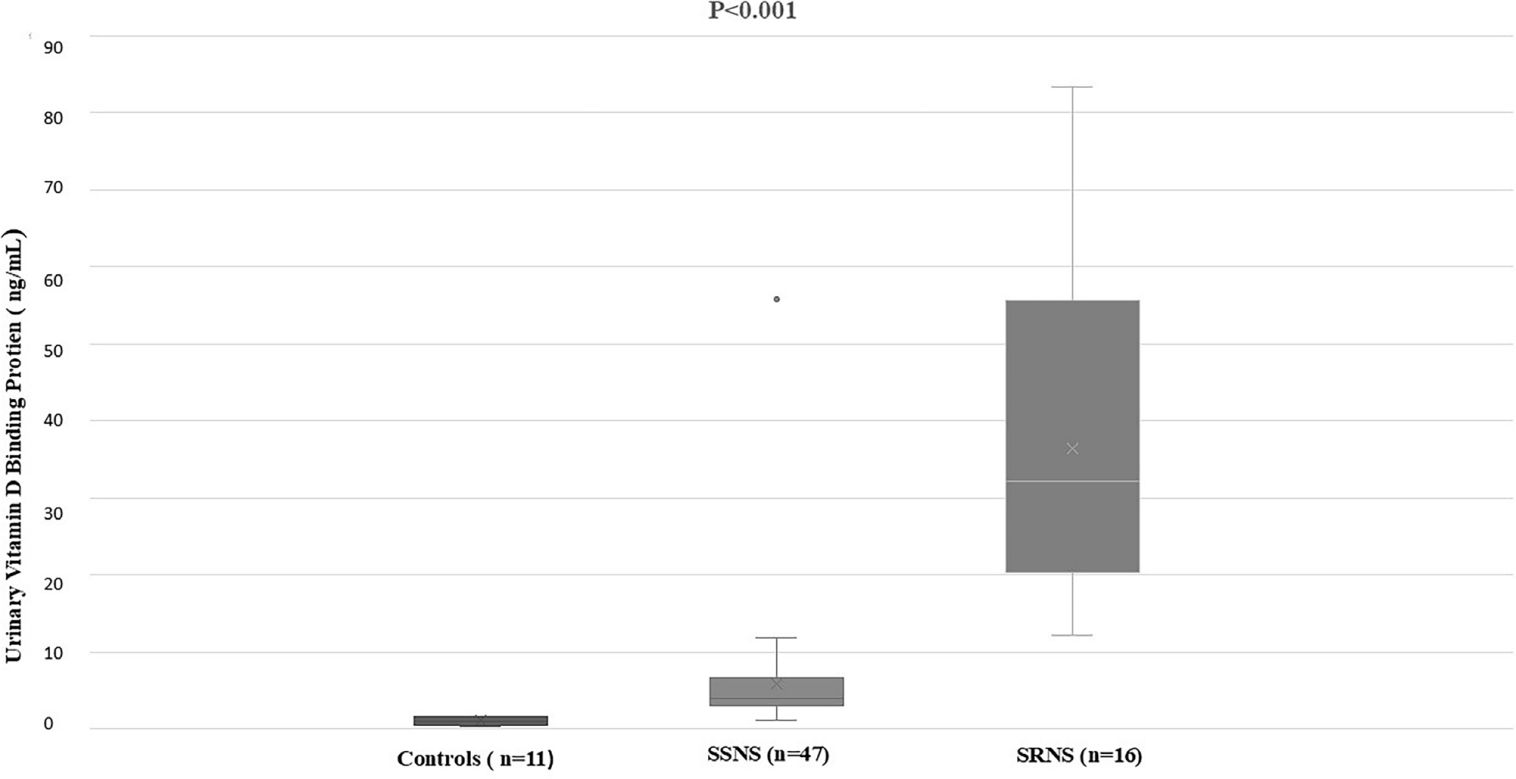
Box plots showing urinary vitamin D binding protein levels in controls and patients (n: number of cases, SSNS: steroid sensitive nephrotic syndrome, SRNS: steroid resistant nephrotic syndrome (short horizontal lines within boxes show median values and vertical lines below and above the boxes represent minimum and maximum values).

**Figure 2:**
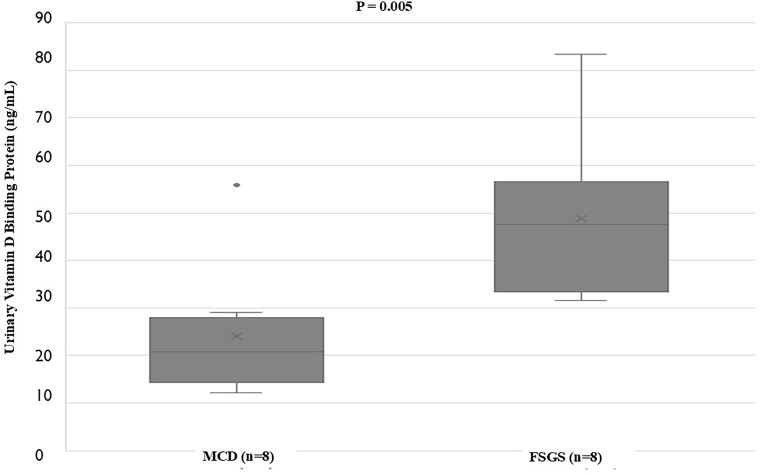
Box plots showing urinary vitamin D binding protein levels in MCD (minimal change disease) and FSGS (focal segmental glomerulosclerosis) cases of steroid resistant nephrotic syndrome (n: number of cases; short horizontal lines within the boxes show median values and vertical lines below and above the boxes represent minimum and maximum values).

The baseline profile of cases and controls is shown in Table 1. Cases of FENS, RNS, and SRNS had significantly lower mean serum 25 (OH) D and serum albumin and significantly higher cholesterol and Upr/Cr levels than controls (P < 0.001). uVDBP values were normalized with urinary creatinine and both uVDBP and uVDBP/cr levels followed similar patterns and were significantly increased in cases than in controls (P < 0.001). The levels showed progressive significant rise across FENS, RNS, and SRNS. The data from FENS and RNS were grouped together as SSNS and compared with SRNS and controls (Table 2). Mean serum 25 (OH) D levels were comparable between SSNS and SRNS, but significantly lower than controls. Median values of Upr/Cr in SSNS and SRNS were similar and significantly higher than controls. Median uVDBP level was significantly increased in SRNS than in SSNS and controls (Figure 1). In SRNS, the median uVDBP value was significantly increased in children with FSGS compared to MCD (47.5 ng/mL vs 20.8 ng/mL, P = 0.005) (Figure 2).

**Table 1:** Basic characteristics and biochemical parameters of study subjects

Parameters	Controls n = 11	FENS n = 23	RNS n = 24	SRNS n = 16	P
Age (years)	4 (2,5)	4 (2,7)	3.5 (2,7)	4.5 (3,6.7)	0.701^a^
Gender Male Female	5 (45.5%) 6 (54.5%)	15 (65.2%) 8 (34.8%)	16 (66.7%) 8 (33.3%)	7 (43.8%) 9 (56.2%)	0.356^b^
Body mass index (kg/m^2^)	15.2 ± 2.1	16.5 ± 2.9	16.8 ± 2.4	17.0 ± 3.2	0.338^c^
Systolic blood pressure (mmHg)	96.5 ± 5.9	100.7 ± 6.5	98.0 ± 7.7	99.6 ± 7.2	0.369^c^
Diastolic blood pressure (mmHg)	59.0 ± 10.4	62.2 ± 6.5	58.9 ± 7.6	63.5 ± 10.1	0.272^c^
eGFRcr (mL/1.73m^2^/min)	100.8 ± 23.7	96.3 ± 28.1	99.9 ± 33.8	99.4 ± 29.9	0.967^c^
Serum albumin (g/dL)	4.1 ± 0.3	2.3 ± 0.5	2.2 ± 0.6	2.0 ± 0.6	<0.001^c^
Serum cholesterol l(mg/dL)	139.9 ± 8.2	371.5 ± 106.4	398.4 ± 146.7	414.5 ± 139.6	<0.001^c^
Blood urea (mg/dl)	24 (19,31)	25 (22,32)	35.5 (21.7,50.2)	29.5 (15.7,47.2)	0.182^a^
Serum creatinine (mg/dL)	0.5 ± 0.08	0.6 ± 0.17	0.6 ± 0.18	0.6 ± 0.11	0.541^c^
Serum sodium (mEq/L)	136.7 ± 2.8	136 ± 4.1	136.7 ± 3.4	136.7 ± 2.9	0.905^c^
Serum potassium (mEq/L)	4.4 ± 0.4	4.2 ± 0.5	4.4 ± 0.4	4.6 ± 0.8	0.298^c^
Serum calcium (mg/dL)	8.6 ± 0.5	8.5 ± 0.8	8.6 ± 0.7	8.3 ± 0.6	0.670^c^
Serum phosphate (mg/dL)	4.9 ± 0.3	4.6 ± 0.7	4.9 ± 0.8	5.0 ± 0.7	0.409^c^
Alkaline phosphatase (U/L)	207.9 ± 58.0	199.1 ± 82.3	191.5 ± 65.3	190.8 ± 38.0	0.892^c^
Serum vitamin 25 (OH) D (ng/mL)	23.2 ± 11.7	5.6 ± 1.1	6.1 ± 1.3	5.2 ± 1.0	<0.001^c^
Urine protein/creatinine ratio (mg/mg)	0.2 (0.2,0.3)	4.9^d^ (4.0,8.8)	8.0^d^ (5.3,16.8)	9.9^d^ (6.0,18.5)	<0.001^a^
Urinary vitamin D binding protein (ng/mL)	1.1 (0.6,1.6)	3.1^d^ (2.4,3.7)	5.7^d^ (4.0,7.9)	32.2^d^ (20.4,55.7)	<0.001^a^
Urinary vitamin D binding protein/ creatinine (ng/mg)	1.4 (0.9,2)	5.7^d^ (3.5,8.1)	10.3^d^ (4.2,19.0)	85.8^d^ (45,141)	<0.001^a^

Abbreviations – n: number of subjects; eGFRcr: Estimated glomerular filtration rate creatinine; FENS: First episode nephrotic syndrome; RNS: Relapsing nephrotic syndrome; SRNS: Steroid resistant nephrotic syndrome.

Notes – a: Kruskal Wallis test

b: Chi-square test

c: Analysis of variance

d: Mann-Whitney U test.

**Table 2:** Biochemical and urinary parameters of controls, steroid-sensitive and steroid-resistant nephrotic syndrome patients

Parameters	Controls n =1 1	SSNS n = 47	SRNS n = 16	P value
Serum calcium (mg/dL)	8.6 ± 0.50	8.6 ± 0.79	8.3 ± 0.68	0.511^a^
Serum phosphate (mg/dL)	4.9 ± 0.34	4.8 ± 0.79	5.0 ± 0.71	0.532^a^
Alkaline phosphatase (u/L)	207 ± 58.0	195.3 ± 73.4	190.8 ± 38.0	0.792^a^
Serum 25 (OH) D (ng/mL)	23.2 ± 11.7	5.8 ± 1.2	5.2 ± 1.0	<0.001^a^
Urine protein creatinine ratio (mg/mg)	0.20 (0.20,0.30)	6.9^c^ (4.2,11.6)	9.9^c^ (6.0,18.5)	<0.001^b^
Urinary vitamin D binding protein (ng/mL)	1.07 (0.61,1.6)	4.0^c^ (3.0,6.6)	32.2^c^ (20.4,55.7)	<0.001^b^
Urinary vitamin D binding protein/ creatinine (ng/mg)	1.47 (0.97,2.0)	6.6^c^ (4.1,14.0)	85.8^c^ (45.0,141.9)	<0.001^b^

Abbreviations – n: number of subjects; SSNS: Steroid sensitive nephrotic syndrome; SRNS: Steroid resistant nephrotic syndrome.

Notes – a: Analysis of variance

b: Kruskal Wallis test

c: Mann Whitney U test.

The uVDBP and 25 (OH) vitamin D assay were repeated in 17 patients [13 SSNS and 4 SRNS] during remission, and analyzed as paired sample observations. The uVDBP showed significant decrease in remission (1.2 ng/mL, IQR 0.9, 1.58) compared to active phase (5.05 ng/mL, IQR 3.2,10.1, P < 0.001). The 25(OH) vitamin D showed significant increase in remission (26.9 ± 16.1 ng/mL) in comparison to active nephrotic syndrome (6.30 ± 1.61, P < 0.001).

**Figure 3:**
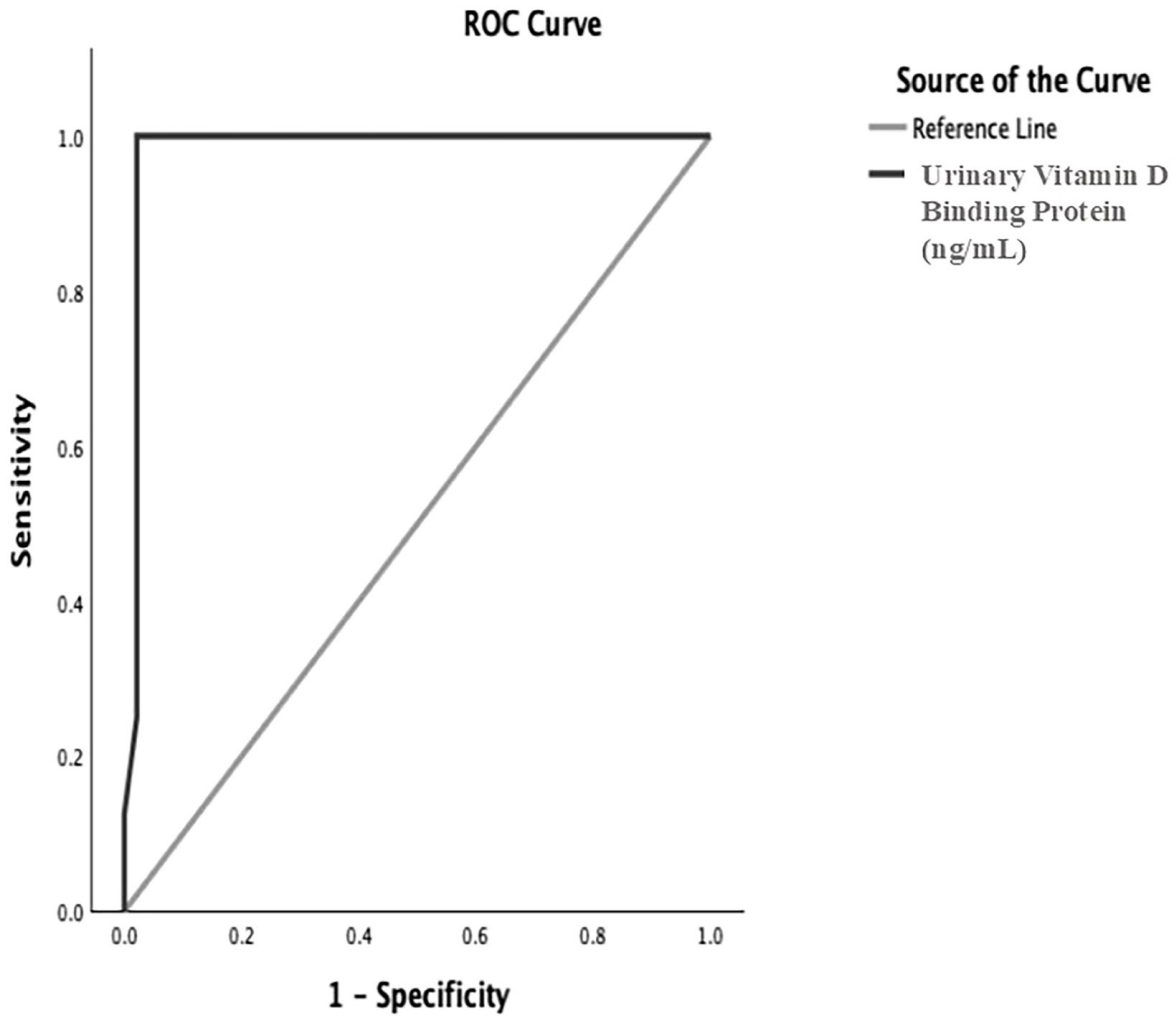
Receiver operating characteristic curve for urinary vitamin D binding protein level to distinguish steroid-resistant from steroid-sensitive nephrotic syndrome.

In addition, a 12-ng/mL cut-off of uVDBP had a sensitivity and specificity of 100% and 97.9% respectively, and AUC of 0.983, P < 0.001 (95% confidence interval 0.949–1.017) to distinguish SRNS from SSNS (Figure 3). The uVDBP showed significant positive correlation with uPr/Cr (r = 0.441, P < 0.001) and negative correlation with serum albumin (r = −0.303, P = 0.016). No relationship of uVDBP with other parameters such as serum (25 OH) vitamin D, cholesterol, and eGFRcr was observed.

## Discussion

Mean 25(OH) vitamin D levels were significantly lower in all the sub-groups of nephrotic syndromes than in controls, as were in active disease compared to remission. The level is reduced in active disease because of the increased urinary excretion of 25 (OH) vitamin D^14,15^. In addition, Yousefichaijan et al.^16^ reported low vitamin D levels (<10 ng/mL) in 17% of steroid sensitive disease, 83% of steroid dependent disease, and 70% of SRNS. Charan et al.^17^ also reported significantly lower serum 25(OH) vitamin D levels and increased uVDBP levels in first episode nephrotic syndrome patients than controls. However, it appears that nephrotic syndrome patients need vitamin D supplementation, especially when they are receiving corticosteroids. Other authors have also reported similar observation in their studies^18^.

Urinary VDBP was normalized with urinary creatinine and both provided similar results. uVDBP showed significantly progressive rises across FENS, RNS, and SRNS cases (P < 0.001). SRNS had significantly higher uVDBP excretion than SSNS, which could be due to the protracted course and chronic tubular injury in SRNS. Higher levels of urinary VDBP excretion in SRNS than in SSNS have also been reported by other authors^13,19,20^. A possible explanation for nephrotic syndrome patients having significantly increased uVDBP is that it is easily filtered by the glomerulus due to its low molecular weight. However, its reabsorption from kidney tubules depends on functional and structural morphology of megalin and cublin receptors in tubules, which may be altered in severe lesions like FSGS. Therefore, cases of SRNS with FSGS histology had significantly higher uVDBP excretion than MCD. Again, this is because of the fact that chronic parenchymal and tubular damage in FSGS may contribute to higher levels of uVDBP in these patients, which is independent of urinary protein excretion. In a previous experimental animal study, uVDBP was reported as a biomarker of interstitial injury and glomeruli fibrosis^21^.

uVDBP showed significant reduction during remission. By contrast, Bennett et al.^19^ reported no significant difference in uVDBP in active and remission disease phases in both SSNS and SRNS. Another study observed no significant variation in uVDBP levels during proteinuric and non-proteinuric cases of SSNS^20^. Authors also reported unchanged values of uVDBP in SRNS patients treated with and without calcineurin inhibitor therapy. Our SRNS patients also received calcineurin inhibitor, but there were only 4, and the remaining 13 cases of remission belonged to the SSNS group. Therefore, there were no patients with persistently high uVDBP level during remission in our study.

To differentiate SRNS from SSNS patients, at a uVDBP cut-off value of 12 ng/mL had sensitivity of 100%, specificity 97.9%, and AUC of 0.983. Other studies have reported lower sensitivity (80–89%), specificity (78.6–84.4%), and AUC (0.78–0.909) at different cut-off levels. Thus, present study showed better ability to distinguish between SRNS to SSNS.

Urinary VDBP correlated positively with uPr/Cr (r = 0.441, P < 0.001) and negatively with serum albumin (r = –303, P = 0.016). This is because of increased uVDBP levels in active cases of nephrotic syndrome, who had heavy proteinuria and hypoalbuminemia. Charan et al.^17^ found a positive correlation of uVDBP with uPr/Cr (r = 0.51, P = 0.004), and of serum VDBP with serum albumin (r = 0.37, P < 0.05). Further, we failed to find a correlation between urinary VDBP and eGFRcr. This was probably due to the fact that none of our cases had kidney function derangement and their eGFRcr values were within normal range. Contrary to our findings, previous studies have reported negative correlations of uVDBP with eGFRcr (r = –0.263 to –0.76), which leads to the conclusion that levels of uVDBP show a rising trend with decreasing kidney function^13,19,20^.

The strength of study is that it was conducted as a prospective longitudinal observation with a relatively good sample size. However, there are certain limitations. Firstly, parameters, especially uVDBP, could not be repeated in all the cases during remission, as the performance of the test with single ELISA kit is limited. Secondly, a smaller number of patients with MCD and FSGS in SRNS patients were enrolled during the study period. Therefore, further studies with larger sample sizes are needed to establish the ability of uVDBP to discriminate between the MCD and FSGS subtypes of SRNS, as the latter do not respond to steroids and kidney function may deteriorate during follow-up.

## Conclusion

Urinary VDBP level was increased in active nephrotic syndrome and could reliably discriminate steroid-resistant from steroid-sensitive cases. uVDBP levels were also higher in biopsy proven FSGS than in MCD subtypes of SRNS.

## Data Availability

The datasets generated and/or analyzed during the current study are not publicly available due to ethical restrictions, but are available from the corresponding author upon reasonable request.
