# Supplementary material for: Urinary vitamin D binding protein levels in children with idiopathic nephrotic syndrome: a biomarker differentiating steroid sensitive from steroid resistant nephrotic syndrome
Source: J Bras Nefrol. 2025 Nov 7;48(1):e20250094. doi: 10.1590/2175-8239-JBN-2025-0094en (PMC12700445; doi:10.1590/2175-8239-JBN-2025-0094en)
Supplement: Translation

# Translation: Níveis de proteína de ligação à vitamina D urinária em crianças com síndrome nefrótica idiopática: um biomarcador que diferencia síndrome nefrótica sensível a esteroides da forma resistente a esteroides

**DOI:** 10.1590/2175-8239-JBN-2025-0094pt

**Authors:** Anup K. Chaudhary, Om P. Mishra, Ketki Khandhadiya, Surendra P. Mishra, Akash Mishra, Ashok Singh, Priyanka Dua

**Affiliations:** 1 Heritage Institute of Medical Sciences, Department of Pediatrics, Varanasi, Índia.; 2 Heritage Institute of Medical Sciences, Department of Biochemistry, Varanasi, Índia.; 3 Banaras Hindu University, Institute of Medical Sciences, Department of Biochemistry, Varanasi, Índia.; 4 Banaras Hindu University, Institute of Medical Sciences, Centre of Biostatistics, Varanasi, Índia.

**Keywords:** Síndrome Nefrótica Idiopática da Infância, Síndrome Nefrótica Corticossensível, Síndrome Nefrótica Resistente a Esteroides, Proteína de Ligação à Vitamina D Urinária

## Abstract

**Introdução:**

Objetivo principal - avaliar níveis de proteína de ligação à vitamina D urinária (uVDBP) na síndrome nefrótica idiopática. Objetivos secundários - determinar a capacidade da uVDBP de diferenciar síndrome nefrótica resistente a esteroides (SNRE) da sensível a esteroides (SNSE) e, no contexto da SNRE, distinguir doença de lesão mínima (DLM) de glomeruloesclerose segmentar focal (GESF).

**Métodos:**

Estudo com 63 casos: 23 de primeiro episódio (SNPE), 24 recorrentes (SNR) e 16 resistentes a esteroides; 11 indivíduos serviram como controles e 17 estavam em remissão. O kit ELISA foi empregado para analisar uVDBP.

**Resultados:**

Níveis medianos de uVDBP foram maiores em SNPE, SNR, SNRE versus controles (P < 0,001). A mediana foi significativamente maior na SNRE (32,2 ng/ml) em relação à SNSE (4,0 ng/ml; P < 0,001). De forma semelhante, a mediana também foi significativamente maior na GESF (56,6 ng/ml) do que na DLM (20,8 ng/ml; P = 0,005). A uVDBP foi significativamente menor durante a remissão (1,2 ng/ml) versus fase ativa (5,1 ng/ml) da doença (P < 0,001). No valor de corte 12 ng/ml, a uVDBP apresentou maior sensibilidade, especificidade e área sob a curva (100%, 97,9%, 0,983, respectivamente) para distinguir pacientes resistentes daqueles sensíveis a esteroides. Observou-se correlação positiva significativa com relação proteína/creatinina urinária (r = 0,441; P < 0,001) e negativa com albumina sérica (r = −0,303; P = 0,019).

**Conclusão:**

O nível de uVDBP relaciona-se à atividade da doença e pode distinguir resistência de sensibilidade a esteroides, além de diferenciar os subtipos histológicos da SNRE, GESF e DLM.

## Introdução

A síndrome nefrótica é uma doença glomerular comum, com incidência anual de 2 a 7/100.000 em crianças com menos de 16 anos de idade^1^. A população asiática apresenta uma incidência mais elevada (9-16 por 100.000 crianças por ano)^2^. A maioria (85-90%) dos casos de síndrome nefrótica idiopática apresenta uma resposta favorável ao tratamento com corticosteroides, sendo classificada como síndrome nefrótica sensível a esteroides (SNSE). No entanto, 10–15% dos casos não alcançam remissão com o tratamento à base de esteroides e são classificados como síndrome nefrótica resistente a esteroides (SNRE)^3^, com uma proporção significativa (30-50%) progredindo para doença renal em estágio terminal dentro de 10 anos^4,5^. Biomarcadores urinários, como a atividade urinária da N-acetil-β-D-glicosaminidase^6^, a lipocalina associada à gelatinase neutrofílica^7, 8, 9^ e o CD 80^10,11^ têm sido utilizados em crianças com síndrome nefrótica idiopática para distinguir entre as formas sensível e resistente a esteroides da doença. No entanto, a sensibilidade e a especificidade para a capacidade discriminatória desses biomarcadores variam entre os estudos. Os níveis também variam entre dois subtipos histopatológicos comuns da SNRE: doença de lesão mínima (DLM) e glomeruloesclerose segmentar e focal (GESF)^8,12^.

A proteína de ligação à vitamina D (VDBP) é produzida principalmente no fígado. Seu peso molecular é semelhante ao da albumina, sendo, portanto, excretada na urina juntamente com a albumina em pacientes com síndrome nefrótica. Os casos de SNRE apresentam proteinúria intensa e curso prolongado, podendo apresentar maior excreção de VDBP na urina em comparação à SNSE. A biópsia renal constitui um procedimento invasivo necessário para diagnosticar GESF e DLM, as quais apresentam resposta diferente aos esteroides e desfechos de longo prazo distintos.

Os níveis de uVDBP também podem variar entre os dois subtipos, com os casos de GESF apresentando proteinúria mais grave do que os de DLM^8^. Portanto, a VDBP, mensurável por meio de um teste não invasivo, pode servir como biomarcador para diferenciar pacientes resistentes de pacientes sensíveis aos esteroides, bem como entre GESF comprovada por biópsia e DLM, no contexto da SNRE. Este estudo teve como objetivo estimar os níveis de uVDBP na síndrome nefrótica idiopática e analisar sua capacidade de distinguir entre SNRE e SNSE, e entre casos de GESF e DLM.

## Métodos

Trata-se de um estudo prospectivo realizado entre dezembro de 2022 e junho de 2024 em um hospital universitário. O estudo foi aprovado pelo Comitê de Ética Institucional (HIMS/IEC/111/2022, datado de 18.11.2022). Os pais/responsáveis legais forneceram consentimento informado por escrito antes da participação no estudo.

### Participantes do Estudo

Foram incluídos pacientes de ambos os sexos, com idade entre 1 e 18 anos, portadores de síndrome nefrótica idiopática (SNI). Os critérios diagnósticos foram presença de edema, proteinúria [relação proteína/creatinina urinária (RPCU) > 2 mg/mg] e redução do nível sérico de albumina (albumina sérica < 3 g/dL). Os critérios de exclusão foram a presença de síndrome nefrótica congênita ou infantil, infecção do trato urinário concomitante, síndrome nefrótica de etiologias secundárias, injúria renal aguda ou doença renal crônica, malformações congênitas do rim e do trato urinário e transplante renal.

O cálculo do tamanho da amostra foi baseado no estudo de Choudhary et al.^13^ sobre uVDBP em pacientes com síndrome nefrótica. Os valores médios e de DP foram 701,12 ± 372,0, 252,87 ± 66,34 e 34,7 ± 14,1 ng/ml em SNRE, SNSE e controles, respectivamente. Considerando uma diferença mínima de 218,1 ng/ml na média a ser detectada entre os grupos, um DP máximo de 372, um nível de significância de 5% e um poder de 90%, o número mínimo de casos a ser incluído em cada grupo foi estimado em 27. Assim, o número total de participantes necessários para o estudo foi de 81. Considerando uma taxa de abandono de 10%, o tamanho total da amostra calculado foi de 81 + 8,1 = 89,1, o qual foi arredondado para 90.

Foram incluídos 63 casos entre diferentes categorias de SNI: SNSE [23 SN de primeiro episódio (SNPE) e 24 síndrome nefrótica recorrente (SNR): 5 recidivas pouco frequentes, 9 recidivas frequentes e 10 dependentes de esteroides] e 16 SNRE. Outras 17 crianças [13 SNSE, (2 SNPE + 11 SNR) e 4 SNRE] também foram incluídas durante a remissão (teste de urina com tira reagente - ausente/traço e/ou relação PCU <0,2 mg/mg por 3 dias consecutivos). Onze crianças saudáveis, na mesma faixa etária, foram selecionadas para servir como controles (o número total de participantes do estudo foi de 63 + 17 + 11 = 91).

### Ensaio Para Proteína de Ligação À Vitamina d Urinária (Uvdbp)

Amostras de urina foram coletadas dos pacientes durante a fase ativa (proteinúrica) da doença em um recipiente estéril, centrifugadas a 5000 rpm por 5 minutos e armazenadas a –20 °C até serem analisadas. A concentração de uVDBP foi estimada utilizando o kit ELISA VDBP Humano disponível comercialmente, fabricado pela ELK (Wuhan) *Biotechnology Co. Ltd.* (http://www.elkbiotech.com). A sensibilidade do kit foi de 0,074 ng/ml (intervalo de referência de detecção de 0,16–10 ng/ml, precisão intraensaio <8% e precisão interensaio <10%).

### Análise Estatística

O software SPSS versão 29.0.2.0. (20) foi utilizado para a análise dos dados. O teste exato de Fisher foi aplicado para a comparação de variáveis categóricas. O teste Análise de Variância (ANOVA) foi utilizado para comparar variáveis com distribuição normal, e o teste de Kruskal Wallis foi aplicado para comparar dados que não seguiam uma distribuição gaussiana. O teste de Bonferroni foi utilizado para a análise post hoc e o teste U de Mann Whitney foi empregado para comparações entre dois grupos. O teste t de Student pareado e o teste de postos sinalizados de Wilcoxon foram utilizados para observações de amostras pareadas, dependendo da distribuição dos dados. Para determinar a sensibilidade, especificidade e área sob a curva da uVDBP na distinção entre SNRE e SNSE, foi gerada a curva ROC (*Receiver Operating Characteristic*). Os coeficientes de correlação (*rho*) de Spearman foram obtidos para a uVDBP em relação a diferentes variáveis, sendo considerado significativo um valor de p < 0,05.

## Resultados

O perfil basal dos casos e controles é apresentado na Tabela 1. Os casos de SNPE, SNR e SNRE apresentaram níveis médios significativamente mais baixos de 25(OH)D sérica e albumina sérica, bem como níveis significativamente mais elevados de colesterol e RPCU, em comparação aos controles (P < 0,001). Os valores de uVDBP foram normalizados com creatinina urinária, e tanto os níveis de uVDBP quanto os de uVDBP/Cr seguiram padrões semelhantes, apresentando aumento significativo nos casos em comparação aos controles (P < 0,001). Os níveis apresentaram um aumento progressivo significativo em SNPE, SNR e SNRE. Os dados de SNPE e SNR foram agrupados como SNSE e comparados com SNRE e controles (Tabela 2). Os níveis médios séricos de 25(OH)D foram comparáveis entre SNSE e SNRE, mas significativamente mais baixos do que nos controles. Os valores medianos de RPCU em SNSE e SNRE foram semelhantes e significativamente mais elevados em relação aos controles. O nível mediano de uVDBP esteve significativamente aumentado na SNRE em comparação à SNSE e aos controles (Figura 1). Na SNRE, o valor mediano de uVDBP esteve significativamente aumentado em crianças com GESF em comparação à DLM (47,5 ng/ml vs. 20,8 ng/ml; P = 0,005) (Figura 2).

**Tabela 1:** Características basais e parâmetros bioquímicos dos participantes do estudo

Parâmetros	Controles n = 11	SNPE n = 23	SNR n = 24	SNRE n = 16	P
Idade (anos)	4 (2;5)	4 (2;7)	3,5 (2;7)	4,5 (3;6,7)	0,701^a^
Sexo Masculino Feminino	5 (45,5%) 6 (54,5%)	15 (65,2%) 8 (34,8%)	16 (66,7%) 8 (33,3%)	7 (43,8%) 9 (56,2%)	0,356^b^
Índice de massa corporal (kg/m^2^)	15,2 ± 2,1	16,5 ± 2,9	16,8 ± 2,4	17,0 ± 3,2	0,338^c^
Pressão arterial sistólica (mmHg)	96,5 ± 5,9	100,7 ± 6,5	98,0 ± 7,7	99,6 ± 7,2	0,369^c^
Pressão arterial diastólica (mmHg)	59,0 ± 10,4	62,2 ± 6,5	58,9 ± 7,6	63,5 ± 10,1	0,272^c^
TFGe-cr (mL/1,73m^2^/min)	100,8 ± 23,7	96,3 ± 28,1	99,9 ± 33,8	99,4 ± 29,9	0,967^c^
Albumina sérica (g/dL)	4,1 ± 0,3	2,3 ± 0,5	2,2 ± 0,6	2,0 ± 0,6	<0,001^c^
Colesterol sérico (mg/dL)	139,9 ± 8,2	371,5 ± 106,4	398,4 ± 146,7	414,5 ± 139,6	<0,001^c^
Ureia no sangue (mg/dl)	24 (19;31)	25 (22;32)	35,5 (21,7;50,2)	29,5 (15,7;47,2)	0,182^a^
Creatinina sérica (mg/dL)	0,5 ± 0,08	0,6 ± 0,17	0,6 ± 0,18	0,6 ± 0,11	0,541^c^
Sódio sérico (mEq/L)	136,7 ± 2,8	136 ± 4,1	136,7 ± 3,4	136,7 ± 2,9	0,905^c^
Potássio sérico (mEq/L)	4,4 ± 0,4	4,2 ± 0,5	4,4 ± 0,4	4,6 ± 0,8	0,298^c^
Cálcio sérico (mg/dL)	8,6 ± 0,5	8,5 ± 0,8	8,6 ± 0,7	8,3 ± 0,6	0,670^c^
Fosfato sérico (mg/dL)	4,9 ± 0,3	4,6 ± 0,7	4,9 ± 0,8	5,0 ± 0,7	0,409^c^
Fosfatase alcalina (U/L)	207,9 ± 58,0	199,1 ± 82,3	191,5 ± 65,3	190,8 ± 38,0	0,892^c^
Vitamina 25(OH)D sérica (ng/ml)	23,2 ± 11,7	5,6 ± 1,1	6,1 ± 1,3	5,2 ± 1,0	<0,001^c^
Relação proteína/creatinina urinária (mg/mg)	0,2 (0,2;0,3)	4,9^d^ (4,0;8,8)	8,0^d^ (5,3;16,8)	9,9^d^ (6,0;18,5)	<0,001^a^
Proteína de ligação à vitamina D urinária (ng/ml)	1,1 (0,6;1,6)	3,1^d^ (2,4;3,7)	5,7^d^ (4,0;7,9)	32,2^d^ (20,4;55,7)	<0,001^a^
Proteína de ligação à vitamina D urinária/creatinina (ng/mg)	1,4 (0,9;2)	5,7^d^ (3,5;8,1)	10,3^d^ (4,2;19,0)	85,8^d^ (45;141)	<0,001^a^

Abreviaturas – n: número de indivíduos; TFGe-cr: Taxa de Filtração Glomerular Estimada pela Creatinina; SNPE: Síndrome Nefrótica de Primeiro Episódio; SNR: Síndrome Nefrótica Recorrente; SNRE: Síndrome Nefrótica Resistente a Esteroides.

Notas – a: Teste de Kruskal Wallis

b: Teste do Qui-quadrado

c: Análise de variância

d: Teste U de Mann-Whitney.

**Tabela 2:** Parâmetros bioquímicos e urinários de controles, pacientes com síndrome nefrótica sensível a esteroides e pacientes com síndrome nefrótica resistente a esteroides

Parâmetros	Controles n = 11	SNSE n = 47	SNRE n = 16	Valor de P
Cálcio sérico (mg/dL)	8,6 ± 0,50	8,6 ± 0,79	8,3 ± 0,68	0,511^a^
Fosfato sérico (mg/dL)	4,9 ± 0,34	4,8 ± 0,79	5,0 ± 0,71	0,532^a^
Fosfatase alcalina (u/L)	207 ± 58,0	195,3 ± 73,4	190,8 ± 38,0	0,792^a^
25 (OH) D sérica (ng/ml)	23,2 ± 11,7	5,8 ± 1,2	5,2 ± 1,0	<0,001^a^
Relação proteína/creatinina urinária (mg/mg)	0,20 (0,20;0,30)	6,9^c^ (4,2;11,6)	9,9^c^ (6,0;18,5)	<0,001^b^
Proteína de ligação à vitamina D urinária (ng/ml)	1,07 (0,61;1,6)	4,0^c^ (3,0;6,6)	32,2^c^ (20,4;55,7)	<0,001^b^
Proteína de ligação à vitamina D urinária/creatinina (ng/mg)	1,47 (0,97;2,0)	6,6^c^ (4,1;14,0)	85,8^c^ (45,0;141,9)	<0,001^b^

Abreviaturas – n: número de indivíduos; SNSE: síndrome nefrótica sensível a esteroides; SNRE: síndrome nefrótica resistente a esteroides.

Notas – a: Análise de variância

b: Teste de Kruskal Wallis

c: Teste U de Mann Whitney.

**Figura 1:** Diagramas de caixa mostrando os níveis de proteína de ligação à vitamina D urinária em controles e pacientes (n: número de casos, SNSE: síndrome nefrótica sensível a esteroides, SNRE: síndrome nefrótica resistente a esteroides); as linhas horizontais curtas dentro das caixas representam os valores medianos, enquanto as linhas verticais abaixo e acima das caixas representam os valores mínimos e máximos.

**Figura 2:** Diagramas de caixa mostrando os níveis de proteína de ligação à vitamina D urinária em casos de DLM (doença de lesão mínima) e GESF (glomeruloesclerose segmentar e focal) da síndrome nefrótica resistente a esteroides (n: número de casos; as linhas horizontais curtas dentro das caixas mostram os valores medianos e as linhas verticais abaixo e acima das caixas representam os valores mínimos e máximos).

O ensaio para detecção de uVDBP e vitamina D 25(OH) foi repetido em 17 pacientes (13 com SNSE e 4 com SNRE) durante a remissão e analisado como observações de amostras pareadas. A uVDBP apresentou uma redução significativa na remissão (1,2 ng/ml; IIQ 0,9; 1,58) em comparação à fase ativa (5,05 ng/ml; IIQ 3,2; 10,1; P < 0,001). A vitamina D 25(OH) apresentou um aumento significativo na remissão (26,9 ± 16,1 ng/ml) em comparação à síndrome nefrótica ativa (6,30 ± 1,61; P < 0,001).

Além disso, um ponto de corte de 12 ng/ml de uVDBP apresentou sensibilidade e especificidade de 100% e 97,9%, respectivamente, e uma AUC de 0,983; P < 0,001 (intervalo de confiança de 95%: 0,949–1,017) para distinguir SNRE de SNSE (Figura 3). A uVDBP apresentou correlação positiva significativa com a RPCU (r = 0,441; P < 0,001) e correlação negativa com a albumina sérica (r = –0,303; P = 0,016). Não foi observada nenhuma relação entre uVDBP e outros parâmetros, como vitamina D sérica (25 OH), colesterol e TFGe-cr.

**Figura 3:** Curva ROC (*Receiver Operating Characteristic*) para o nível de proteína de ligação à vitamina D urinária para distinguir a síndrome nefrótica resistente a esteroides da síndrome nefrótica sensível a esteroides.

## Discussão

Os níveis médios de vitamina D 25(OH) foram significativamente mais baixos em todos os subgrupos das síndromes nefróticas em comparação aos controles, assim como na fase ativa da doença em comparação à remissão. Os níveis são reduzidos na doença ativa devido ao aumento da excreção urinária de vitamina D 25(OH)^14,15^. Além disso, Yousefichaijan et al.^16^ relataram baixos níveis de vitamina D (<10 ng/ml) em 17% dos casos de doença sensível a esteroides, 83% dos casos de doença dependente de esteroides e 70% dos casos de SNRE. Charan et al.^17^ também relataram níveis significativamente mais baixos de vitamina D 25(OH) sérica e níveis aumentados de uVDBP em pacientes com síndrome nefrótica de primeiro episódio em comparação aos controles. No entanto, parece que os pacientes com síndrome nefrótica necessitam de suplementação de vitamina D, especialmente quando em uso de corticosteroides. Outros autores também relataram observações semelhantes em seus estudos^18^.

A VDBP urinária foi normalizada pela creatinina urinária e ambas apresentaram resultados semelhantes. A uVDBP apresentou aumentos significativamente progressivos entre os casos de SNPE, SNR e SNRE (P < 0,001). A SNRE apresentou uma excreção de uVDBP significativamente mais elevada do que a SNSE, o que pode ser devido ao curso prolongado e à lesão tubular crônica observada na SNRE. Níveis mais elevados de excreção urinária de VDBP na SNRE, em comparação à SNSE, também foram relatados por outros autores^13,19,20^. Uma possível explicação para o aumento significativo da uVDBP em pacientes com síndrome nefrótica é o fato de ela ser facilmente filtrada pelo glomérulo devido ao seu baixo peso molecular. No entanto, sua reabsorção nos túbulos renais depende da morfologia funcional e estrutural dos receptores megalina e cublina nos túbulos, que podem ser alterados em lesões graves, como a GESF. Portanto, os casos de SNRE com histologia de GESF apresentaram excreção de uVDBP significativamente mais elevada do que os casos de DLM. Novamente, isso se deve ao fato de que os danos crônicos ao parênquima e aos túbulos renais na GESF podem contribuir para níveis mais elevados de uVDBP nesses pacientes, o que é independente da excreção urinária de proteínas. Em um estudo experimental prévio em animais, a uVDBP foi relatada como um biomarcador de lesão intersticial e fibrose glomerular^21^.

A uVDBP apresentou redução significativa durante a remissão. Em contrapartida, Bennett et al.^19^ não relataram diferença significativa nos níveis de uVDBP nas fases ativa e de remissão da doença, tanto na SNSE quanto na SNRE. Outro estudo não observou variação significativa nos níveis de uVDBP durante casos proteinúricos e não proteinúricos de SNSE^20^. Os autores também relataram valores inalterados de uVDBP em pacientes com SNRE tratados com e sem inibidores da calcineurina. Nossos pacientes com SNRE também receberam inibidores da calcineurina, mas havia apenas quatro, sendo que os 13 casos restantes de remissão pertenciam ao grupo com SNSE. Portanto, não houve pacientes com níveis persistentemente elevados de uVDBP durante a remissão em nosso estudo.

Para diferenciar pacientes com SNRE daqueles com SNSE, um valor de corte de 12 ng/ml para uVDBP apresentou sensibilidade de 100%, especificidade de 97,9% e AUC de 0,983. Outros estudos relataram sensibilidade (80-89%), especificidade (78,6-84,4%) e AUC (0,78-0,909) mais baixas em diferentes níveis de corte. Assim, o presente estudo demonstrou melhor capacidade de distinguir entre SNRE e SNSE.

A VDBP urinária apresentou correlação positiva com a RPCU (r = 0,441; P < 0,001) e negativa com a albumina sérica (r = –303; P = 0,016). Isso se deve ao aumento dos níveis de VDBP urinária em casos ativos de síndrome nefrótica, caracterizados por proteinúria intensa e hipoalbuminemia. Charan et al.^17^ constataram uma correlação positiva entre uVDBP e RPCU (r = 0,51; P = 0,004) e entre VDBP sérica e albumina sérica (r = 0,37; P < 0,05). Além disso, não foi possível constatar uma correlação entre a VDBP urinária e a TFGe-cr. Isso provavelmente se deve ao fato de que nenhum dos nossos casos apresentava alteração da função renal e seus valores de TFGe-cr estavam dentro da faixa de normalidade. Ao contrário de nossos achados, estudos anteriores relataram correlações negativas entre uVDBP e TFGe-cr (r = –0,263 a –0,76), o que leva à conclusão de que os níveis de uVDBP apresentam uma tendência de aumento com a diminuição da função renal^13,19,20^.

O ponto forte do estudo reside no fato de ter sido conduzido como uma observação longitudinal prospectiva com uma amostra de tamanho relativamente adequado. No entanto, existem certas limitações. Em primeiro lugar, não foi possível repetir os parâmetros, especialmente a uVDBP, em todos os casos durante a remissão, em razão das limitações do desempenho do teste com um único kit ELISA. Em segundo lugar, durante o período do estudo, foi incluído um número reduzido de casos de DLM e GESF entre os pacientes com SNRE. Portanto, são necessários estudos adicionais com amostras maiores para estabelecer a capacidade da uVDBP de distinguir entre os subtipos DLM e GESF da SNRE, uma vez que estes últimos não respondem aos esteroides e a função renal pode deteriorar-se durante o acompanhamento.

## Conclusão

O nível de VDBP urinária esteve aumentado na síndrome nefrótica ativa e foi capaz de distinguir de forma confiável os casos resistentes a esteroides dos casos sensíveis a esteroides. Os níveis de uVDBP também foram mais elevados na GESF comprovada por biópsia em comparação à DLM, subtipos da SNRE.

## Data Availability

Os conjuntos de dados gerados e/ou analisados durante o presente estudo não estão disponíveis publicamente devido a restrições éticas, mas podem ser obtidos junto ao autor correspondente mediante solicitação razoável.
